# Lithium alters expression of RNAs in a type-specific manner in differentiated human neuroblastoma neuronal cultures, including specific genes involved in Alzheimer’s disease

**DOI:** 10.1038/s41598-019-54076-3

**Published:** 2019-12-04

**Authors:** Bryan Maloney, Yokesh Balaraman, Yunlong Liu, Nipun Chopra, Howard J. Edenberg, John Kelsoe, John I. Nurnberger, Debomoy K. Lahiri

**Affiliations:** 1 Department of Psychiatry, Stark Neurosciences Research Institute, Indiana University School of Medicine, Indianapolis, USA; 2 Department of Biochemistry and Molecular Biology, Indiana University School of Medicine, Indianapolis, USA; 3 Department of Medical and Molecular Genetics, Indiana University School of Medicine, Indianapolis, USA; 4 Department of Psychiatry, University of California San Diego, La Jolla, CA 92093 USA

**Keywords:** Neurodegeneration, Translational research

## Abstract

Lithium (Li) is a medication long-used to treat bipolar disorder. It is currently under investigation for multiple nervous system disorders, including Alzheimer’s disease (AD). While perturbation of RNA levels by Li has been previously reported, its effects on the whole transcriptome has been given little attention. We, therefore, sought to determine comprehensive effects of Li treatment on RNA levels. We cultured and differentiated human neuroblastoma (SK-N-SH) cells to neuronal cells with all-*trans* retinoic acid (ATRA). We exposed cultures for one week to lithium chloride or distilled water, extracted total RNA, depleted ribosomal RNA and performed whole-transcriptome RT-sequencing. We analyzed results by RNA length and type. We further analyzed expression and protein interaction networks between selected Li-altered protein-coding RNAs and common AD-associated gene products. Lithium changed expression of RNAs in both non-specific (inverse to sequence length) and specific (according to RNA type) fashions. The non-coding small nucleolar RNAs (snoRNAs) were subject to the greatest length-adjusted Li influence. When RNA length effects were taken into account, microRNAs as a group were significantly less likely to have had levels altered by Li treatment. Notably, several Li-influenced protein-coding RNAs were co-expressed or produced proteins that interacted with several common AD-associated genes and proteins. Lithium’s modification of RNA levels depends on both RNA length and type. Li activity on snoRNA levels may pertain to bipolar disorders while Li modification of protein coding RNAs may be relevant to AD.

## Introduction

Lithium (Li) has been used to effectively treat bipolar disorder for more than 60 years^1^. Li may affect cellular signaling processes and promote long-term neuroplasticity^2,3^. Li also seems to have neurotrophic properties affecting cell survival and apoptosis mechanisms^4^, and trace levels may to some extent prevent dementia^5^, suicide^6^, and homicide^7^. The breadth of effects for Li suggest that the metal may be a trace nutrient^8^. In particular, Li levels in drinking water correlated with reduced age-adjusted Alzheimer’s disease (AD) mortality^9^ and incidence^10^. Several investigations of using Li to treat AD or specific symptoms associated with AD are ongoing^11–14^. However, Li toxicity produces Parkinson’s disease (PD) - and AD-like outcomes. Li is currently under active investigation for a broad array of psychiatric and neurological disorders (including AD, Niemann-Pick disease, frontotemporal dementia, and ALS), as well as gastrointestinal disease, neoplasia, and other non-neurological conditions. As of June 11, 2019, the US National Institutes of Health (NIH) lists 141 planned, recruiting, or active clinical trials for Li effects on psychiatric and nervous system disorders, along with an additional 104 for conditions that do not involve the nervous system (http://www.clinicaltrials.gov).

The mechanisms underlying lithium therapeutic activity are still not clear^15,16^. Three principal hypotheses are its action on i) cyclic AMP^17,18^, ii) inositol depletion^19^, and iii) the inhibition of protein kinases, including glycogen synthase kinase, with subsequent activation of the *wnt* neurodevelopmental pathway^20,21^. Li also increases levels of the anti-apoptotic protein Bcl2 in the frontal cortex of rat brain^22^. Li displays general neuroprotective effects, including against excitotoxic lesions^23^ as well as protection against β-amyloid induced cell death^24^. Li increases N-acetyl-aspartate levels in human brain (a measure of neuronal viability) during therapeutic treatment, as measured by magnetic resonance spectroscopy^25^. This increase appears to be related to increases in grey matter volume^26^. Li, similarly to antidepressants, increases hippocampal neurogenesis^27^. Nevertheless, multiple clinical effects of Li are not entirely explained by prevailing theories^28^. Use of neuronally-differentiated pluripotent stem cells from bipolar patients who were responsive to Li vs non-responders revealed that neuronal hyperactivity responded to Li in responders but not in non-responders^29,30^. It should be noted that these cells, even though taken from living patients, represent early developmental stages of central nervous system (CNS) cells. Changes in adult neuronal cells from Li-responsive patients may be related to decreased phosphorylation of collapsin response mediator protein-2 (CRMP2) and increased dendritic spine density^31^.

Since so many pathways are apparently involved in Li treatment of bipolar disorder, and the pathways associated with possible Li treatment of other disorders, such as AD, are yet unknown, a reasonable question is how Li alters the transcriptome. Gene expression changes induced by Li treatment exist in both neuronal cultures and animals^32–35^. However, those reports were array-based assays, not whole transcriptome surveys. If a specific RNA were not *a priori* included on an array, Li effects on it would not be measured. Thus, we performed whole transcriptome sequencing of Li-treated vs. untreated cells. Neuronal cell lines have the advantage of standardization of biochemical characteristics, ease of adaptation to multiple experimental protocols, and simulation of adult CNS cells’ chemical and physiologic characteristics. We found that Li changes expression of multiple small RNA species, particularly non-coding small nucleolar RNAs (snoRNA), in all-trans retinoic acid (ATRA) differentiated human neuroblastoma neuronal (SK-N-SH) culture, but that small RNA species do not all respond to Li stimulation equally.

The snoRNAs may function in multiple neurobiological disorders and symptoms, including catatonia^36^. snoRNA levels differed between schizophrenic and control subjects in the anterior cingulate cortex^37,38^. snoRNA may also play a role in some features of autism^39,40^. Finally, specific snoRNAs undergo progressive changes with age^41^, and snoRNA levels vary with progressing Braak stages in AD^42^. We, therefore, investigated potential connections among Li-perturbed genes and AD-associated genes and proteins. Our results generated interaction networks that can inform future mechanistic research, relevant to AD, bipolar and other neuropsychiatric disorders.

## Results

### Cell culture response

No gross differences in cell morphology or survival were noted.

### Small RNAs, particularly small nucleolar RNAs are over-represented among Li-influenced genes

**Figure 1:**
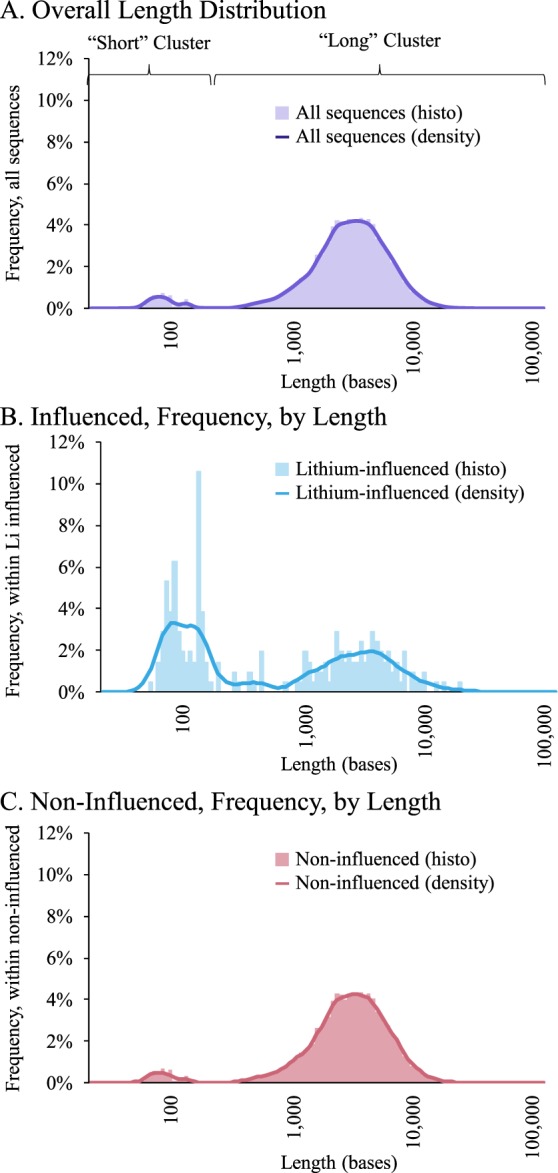
Size distribution of transcriptome, and relative distributions of Li-influenced vs. non-influenced transcriptome, and mean log-lengths of Li-influenced/-non-influenced RNAs. Both histograms and kernel density estimations^44^ of the distributions are shown. (**A**) Sequence frequencies by logarithms of sequence lengths. Log-lengths have a bimodal distribution with parameterized Gaussian mixture model clusters indicated as “Short” and “Long” and specific parameters in Table 1. (**B–C**) Sequences were separated by whether or not expression levels were altered by Li treatment. Relative frequencies within each group (Li-influenced vs. non-influenced) were calculated by dividing counts by each group’s respective total number of sequences and plotted vs log(length). (**B**) Frequency of Li-altered RNAs by length. Line is corresponding kernel density estimation. (**C**) Frequency of non-influenced RNAs by length. Line is corresponding kernel density estimation.

We treated neuronally differentiated human cell cultures with Li and measured relative change vs. vehicle (distilled water)-treated cells in RNA levels, detected by whole-transcriptome sequencing. We found that log lengths of whole transcriptome sequencing had a bimodal distribution. Parameterized Gaussian mixture model^43,44^, cluster centers corresponded to 87 nucleotides (range 22 to 213) and 3047 nucleotides (range 233 to 116,854) in length (Fig. 1A, Table 1). Li significantly altered the expression of 207 RNA species of 15,394 sequenced (Supplementary Table 1) at a false discovery rate (FDR) ≤ 0.2, making Li influence an uncommon event. We did not consider magnitude of influence in our analysis, although the lowest absolute magnitude of change for RNA sequences with FDR ≤ 0.2 was −20% (Supplementary Table 2). Comparing the distribution of altered vs. non-altered sequences indicated that shorter RNA sequences predominated in the Li-influenced group (Fig. 1B), while relative distribution of non-influenced sequence lengths was more similar to the distribution of all sequence lengths (Fig. 1C). Of the 207 Li-influenced RNAs, 103 (49.8%) were in the “short” cluster vs 1031 of 15187 (6.8%) non-influenced RNAs.

**Table 1:** Gaussian mixture model clustering of transcriptome sequence lengths.

Cluster	Center (log)^a^	Minimum (log)	Maximum (log)
Short	87 (1.938)	22 (1.342)	213 (2.328)
Long	3047 (3.484)	233 (2.367)	116,854 (5.068)

^a^Log figures are mean of logs. All analysis was done on logs of lengths.

### RNA species influenced response to Li

To investigate whether the difference simply reflected RNA transcript length or was also influenced by RNA species/type, we used logistic regression to model presence/absence of Li alteration by type of RNA and sequence length. The optimal model derived was “Li influenced ~ log(Length) + RNA Type”. We discovered an odds ratio (OR) of 0.729 +0.226/−0.171 (p ≤ 0.05) for log RNA length vs. Li influence, irrespective of RNA type, i.e., as RNA length decreased, likelihood of Li influence increased.

**Figure 2:**
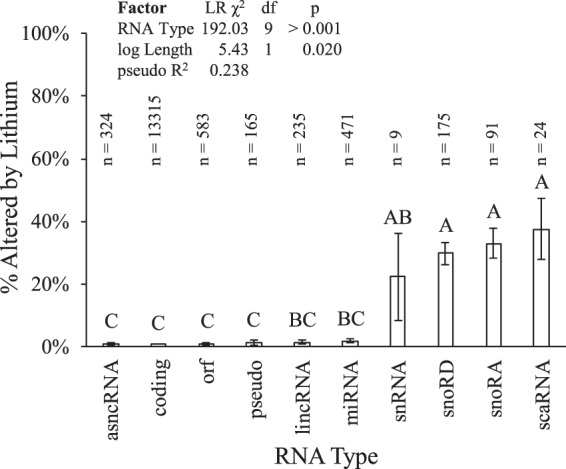
Frequency/likelihood of Li alteration of RNA levels by type and relative distribution of small non-coding RNA species between Li-influenced vs. non-influenced “short” cluster RNAs. Estimated probabilities of alteration of RNA levels by RNA type, taking effect of transcript length into account. Estimated model log odds ratios were compared and pairwise comparison p values adjusted by FDR^45^. RNA types sharing a letter did not differ at p ≤ 0.05. Pseudo R^2^ is Efron’s.

Of greater interest, we found that Li treatment had significantly different effects among RNA types/species, independent of sequence length. Two species (Small NF90 (ILF3) associated RNAs/snaRNA and vault RNA/vtRNA) were each represented in our sample by a single sequence. We excluded both RNA types from further analysis. When compared to overall levels of perturbation across all RNA types (effect coding), levels of 4 types were significantly more likely (OR > 1, p ≤ 0.05) to be altered by Li (Table 2), independently of RNA sequence length; specifically, small Cajal body-specific RNAs (scaRNA), 38% of scaRNA sequences; small nucleolar RNAs, H/ACA box (snoRA), 33% of snoRA sequences; small nucleolar RNAs, C/D box (snoRD), 30% of snoRD sequences; and small nuclear RNAs (snRNA), 22% of snRNA sequences. Levels of 4 types were significantly less likely (OR < 1, p ≤ 0.05) to be altered by Li treatment, specifically microRNA (miRNA), 1.7% of miRNA sequences; uncharacterized open reading frames (orf), 0.9% of orf sequences; protein coding RNA (coding), 0.7% of coding sequences; and antisense non-coding RNA (asncRNA), 0.6% of asncRNA sequences. To distinguish specific differences among the RNA species, we performed pairwise comparisons of ORs by RNA types, adjusted by FDR^45^ and found three overlapping groups (Fig. 2).

**Table 2:** RNA types influenced by Li treatment.

Species^a^	Total	Li Effect		percent ± SE	Logistic Modeling^bc^			Trend^c^.
		altered	not		OR^d^	p	group^e^	
snaRNA^d^	1	0	1	0.00% ± 0.00%	na	na	na	na
asncRNA	324	2	322	0.62% ± 0.44%	0.21 +0.45/−0.17	0.023	C	Down
coding	13315	93	13222	0.70% ± 0.07%	0.28 +0.24/−0.13	<0.001	C	Up
orf	583	5	578	0.86% ± 0.38%	0.29 +0.40/−0.19	0.009	C	Down
pseudo	165	2	163	1.21% ± 0.85%	0.43 +0.92/−0.35	0.215	C	Up
lincRNA	235	3	232	1.28% ± 0.73%	0.45 +0.79/−0.33	0.172	BC	Up
miRNA	471	8	463	1.70% ± 0.60%	0.22 +0.27/−0.13	<0.001	BC	None
snRNA	9	2	7	22.22% ± 13.86%	3.47 +10.71/−2.89	0.110	AB	Up*
snoRD	175	52	123	29.71% ± 3.45%	5.32 +4.76/−2.46	<0.001	A	Up*
snoRA	91	30	61	32.97% ± 4.93%	7.29 +6.03/−3.26	<0.001	A	Up*
scaRNA	24	9	15	37.50% ± 9.88%	9.95 +12.83/−5.76	<0.001	A	Up*
vtRNA^d^	1	1	0	100.00% ± 0.00%	na	na	na	na
Sum	15394	207	15187	1.36%	na	na	na	na

^a^asncRNA: antisense noncoding RNA; coding: protein coding mRNA; lincRNA: long intergenic noncoding RNA; miRNA: micro-RNA; orf: uncharacterized RNA (open reading frame); pseudo: pseudogene RNA; scaRNA: small Cajal body specific RNA; snaRNA: small NF90 (ILF3) associated RNA; snoRA: small nucleolar RNAs, H/ACA box; snoRD: small nucleolar RNAs, C/D boxsnRNA: small nuclear RNA; vtRNA: vault RNA.

^b^Model coded to test the hypothesis of whether or coefficient differs from the mean of groups. It is appropriate for multiple pairwise comparisons.

^c^Derived from multinomial logistic modeling of Type + log(Length) vs. whether Li treatment significantly reduced, elevated, or had no effect on FC transcript levels. “*” indicates difference was significant at p < 0.05.

^d^OR is for effect of RNA type from the model (Li Effect) ~ log(Length) + (RNA Type). ± is 95% confidence intervals. OR marked “*” were significantly different from zero.

^e^Marginal means statistical group, FDR corrected. RNA species sharing letter did not significantly differ in odds of being altered by Li treatment, independent of RNA sequence length effect.

^d^Excluded from logistic model.

We also investigated whether a given RNA species would be more likely to be upregulated or downregulated by Li treatment. We found that snRNA, snoRD, snoRA, and scaRNA were all significantly (p < 0.05) likely to have elevated levels after Li treatment (vs. reduced levels), while other RNA species had no significant effect (Table 3).

**Table 3:** Li-influenced changes in RNA levels of genes implicated in AD.

Gene	Product	Change	Ontologies	AD assoc	Co-exp.	PPI
BMP4	bone morphogenetic protein 4	+51%	Regulation Cell comp.org Cell Proc Developmental Metabolic Processes	^83–85,109^	absent	present
PTMA	prothymosin α	+48%		^110^	present	present
SOX5	SRY box 5	−45%	Regulation Developmental Metabolic Processes	^111^	absent	present
RAB3A	ras-related protein 3A	−44%		^112,113^	present	present
NRXN3	neurexin-3 α	−43%		^88–90^	present	absent
GLIS3	GLIS family zinc finger 3	−42%	Developmental Multicellular Processes	^114,115^	present	present
HS6ST2	heparan sulfate 6-O-sulfotransferase 2	+41%		^116^	present	absent
NMB	neuromedin B	+41%		^117–120^	present	absent
HGF	hepatocyte growth factor	+38%		^121–124^	absent	present
GPRC5B	G-protein coupled receptor family C group 5 member B	−34%	Regulation Cell comp.org Immune Metabolic Proc Multicellular Processes Response to Stimulus	^125^	present	present
YAP1	yes-associated protein 1	−32%	Regulation Metabolic Processes	^126^	present	present
SMAD6	SMAD family member 6	−29%	Regulation Metabolic Processes Response to Stimulus Signaling	^127^	absent	present
GREM2	gremlin 2	−28%		^128^	present	absent
IRS1	insulin receptor substrate 1	−28%	Regulation	^129^	present	present
IGFBP2	insulin-like growth factor binding protein 2	+28%		^130–133^	absent	present
CUX2	cut-like homeobox 2	−27%	Regulation Localization Metabolic Processes	^134^	absent	present
ZWINT	ZW10 interacting kinetochore protein	+27%		^135^	present	present
PPARG	peroxisome proliferator-activated receptor γ	−26%	Regulation Developmental Localization Metabolic Processes Response to Stimulus	^86,87^	present	present

### Li altered levels of specific protein-coding mRNAs and snoRNAs potentially associated with AD

**Figure 3:**
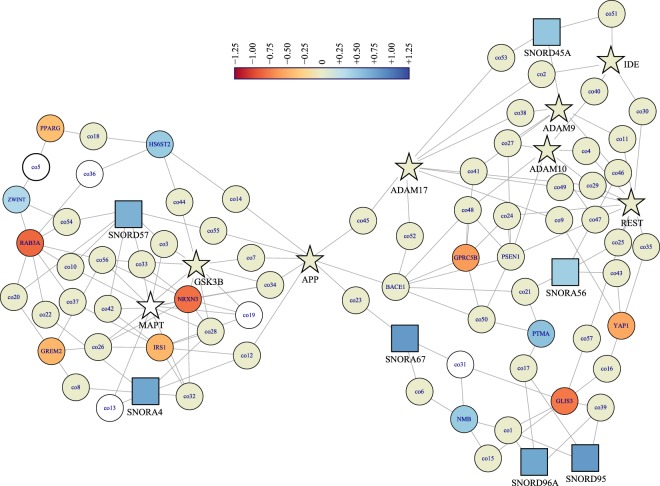
Human hippocampus genetic co-expression network of snoRNA, selected Li-influenced protein coding genes and “core” AD genes. Gene symbols and Li-induced fold-change for snoRNAs, “core” AD genes (Table 5), and selected Li-influenced genes (Table 3) were analyzed for co-expression in human hippocampus by NetworkAnalyst. “Core” AD genes are indicated by star nodes. snoRNAs have square nodes. Color indicates log_2_ fold-change induced by Li treatment, according to legend. White nodes did not appear in our dataset. Additional genes inserted by NetworkAnalyst are in Supplementary Table 3.

**Figure 4:**
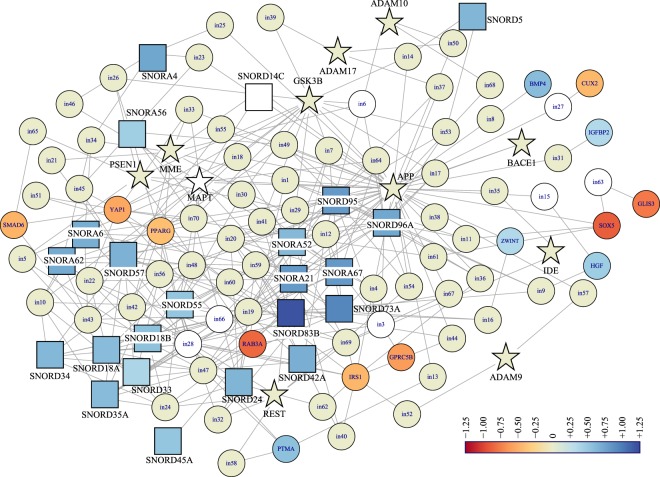
Human hippocampus gene product’s interaction network of snoRNA, selected Li-influenced protein coding genes and “core” AD genes. Gene symbols and Li-induced fold-change for snoRNAs, “core” AD genes (Table 5), and selected Li-influenced genes (Table 3) were analyzed for product interaction in human hippocampus by NetworkAnalyst. This map appears to reveal a multi-target convergence on APP. “Core” AD genes are indicated by star nodes. snoRNAs have square nodes. Color indicates log_2_ fold-change induced by Li treatment, according to legend. White nodes did not appear in our dataset. Additional genes inserted by NetworkAnalyst are in Supplementary Table 4.

Although the majority of Li influence concentrated on small, noncoding RNAs, several protein-coding mRNAs influenced by Li in this study play a role in AD (Table 3). The genes are involved in several Gene Ontology biological functions, predominately biological regulation (GO:0065007) and metabolic processes (GO:0008152). We created two networks (human hippocampus gene co-expression and human hippocampus gene product interaction) using these genes plus a selection of “core” AD-related genes (Table 4). Networks showed several connections between the Li-influenced genes and AD-related genes. However, all such connections were second-degree, i.e., any AD-related gene did not co-express directly with a Li-influenced gene. Instead, a third gene co-expressed with both the AD-related gene and the Li-influenced gene (Fig. 3). On the other hand, when we built the protein-protein network, peroxisome proliferator-activated receptor γ (PPARγ), and SRY-related HMG-box 5 (SOX5) transcription factor directly interacted with the amyloid-β (Aβ) precursor protein (APP), along with SNORD14C (Fig. 4). Paradoxically, none of the snoRNAs associated with Braak staging (Table 5) appeared in either network. Thus, while Li may not directly modify expression of the better-known AD genes, a multi-target effect converging on APP could explain why micro-dosing may be effective, since APP could be the recipient of multiple outcome chains of Li activity.

**Table 4:** “Core” AD genes compared to Li-influenced coding and snoRNA sequences.

Gene^a^	Product^b^	Functions
APP	amyloid β precursor protein	parental protein of neurotoxic/amyloidogenic Aβ peptide and neurotrophic sAPPα
BACE1	β-secretase 1	rate-limiting enzyme in production of Aβ from APP
ADAM9	ADAM metallopeptidase domain 9	α-secretase, non-amyloidogenic cleaving enzyme for APP
ADAM10	ADAM metallopeptidase domain 10	α-secretase, non-amyloidogenic cleaving enzyme for APP
ADAM17	ADAM metallopeptidase domain 17	α-secretase, non-amyloidogenic cleaving enzyme for APP
PSEN1	presenilin 1	critical constituent of γ-secretase complex, which completes APP cleavage processing
MME	membrane metalloendopeptidase	clearance enzyme for Aβ
IDE	insulin degrading enzyme	clearance enzyme for Aβ
MAPT	microtubule-associated protein τ	primary protein constituent of intraneuronal tangles typical of AD
GSK3B	glycogen synthase kinase 3β	primary kinase contributing to pro-tangle phosphorylation of microtubule-associated protein τ
REST	RE1-silencing transcription factor	transcription repression, varies significantly with age

^a^No genes in this table were significantly influenced by Li treatment in our data.

^b^Product name as given in NCBI *Gene* database.

**Table 5:** Li-influenced snoRNA sequences^a^ associated with Braak staging^42^.

Name	Li-induced Change
SCARNA22	+41.7%
SCARNA3	+92.9%
SCARNA6	+52.0%
SNORA37	+69.6%
SNORD104	+83.9%
SNORD46	+48.2%
SNORD60	+42.1%
SNORD94	+63.2%

## Discussion

We demonstrated two effects of Li treatment on the whole RNA transcriptome of a human neuronal cell line. First, Li treatment is more likely to perturb levels of a given RNA inversely to its sequence length, regardless of RNA type. Second, and more importantly, specific RNA type exerts a significant influence on whether or not Li treatment perturbs its levels. That is, snoRNAs of three types (snoRD, snoRA, and scaRNA) are all significantly more likely to be perturbed while miRNA is significantly less likely to be perturbed. It is particularly noteworthy that miRNAs, although often of the same approximate length as snoRNAs, were significantly less likely to have levels perturbed by Li treatment.

SnoRNAs act as a guide in processing nuclear and ribosomal RNAs^46,47^. The functions of snoRNAs may extend to regulation of alternative splicing, gene silencing, chromatin modification and may even exhibit miRNA-like function^48–54^. Understanding the role of snoRNAs in behavior and disease conditions is also emerging. Prader-Willi syndrome is a genetic disorder characterized by intellectual disabilities, hyperphagia, obesity, sleep disorders and behavioral problems; the mechanism involves a paternal deletion of chromosomal region 15q11-q13 harboring snoRNAs SNORD115 (elevated in this study by +84%, FDR = 0.05) and SNORD116 (elevated in this study by +74%, FDR = 0.01). Mice with a deletion of the Snord116 repeat cluster (Snord116del) show a diurnal disruption in feeding behaviors. Brain transcripts associated with Snord116del show upregulation of diurnally regulated genes, particularly Mtor (mammalian target of rapamycin), Crebp (Creb-binding protein), and Igf2r (insulin growth factor receptor) as well as clock genes, with accompanying greater body lean-ness and increased lipid oxidation during daylight hours^55^. Clock genes appear to be disrupted in bipolar disorder as well^56,57^, although the relationship is complex^58^; there is evidence implicating circadian rhythm changes in the response to Li^59^. SNORD115 also regulates maturation of serotonin receptor 2 C (5HT2C) by alternative splicing^60,61^.

It is notable that snoRNAs are active in chromatin modification. Genome wide association studies (GWAS) implicate histone methylation pathways as the most likely targets of snoRNA chromatin modification^62^. In addition, alternative splicing is likely to be important in bipolar disorder^63^.

Li influences snoRNA processing in a yeast model^64^, which may be enlightening to understand potential mechanism in mammalian cells. Specifically, Li appears to inhibit the bisphosphate nucleotidase Hal2p. This results in accumulation of adenosine 3′,5′-bisphosphate (pAp), which is normally broken down by Hal2p, and of pre-snoRNAs. Accumulation of pAp inhibits activities of RNases necessary for processing of snoRNAs. The human homologue for Hal2p is 3′(2′), 5′-bisphosphate nucleotidase 1, or PAP phosphatase (BPNT1). Li inhibits BPNT1 activity in crude human brain extracts^65^. Paradoxically, BPNT1 protein levels are *reduced* in the frontal cortex of bipolar patients^65^. Thus, the specific anti-bipolar activity of Li is unlikely to be a single mechanism.

Although Li influence on miRNA was very limited, some of the specific miRNAs with significantly altered expression play roles in neuropsychiatric disorders. For example, miR-10b was elevated 44% by Li treatment. Elevated expression of miR-10b, in cerebrospinal fluid (CSF), can serve as a diagnostic feature for Parkinson’s disease (PD)^66^. Paradoxically, miR-10b levels are reduced in PD brain, and greater levels of miR-10b associate with later age of motor onset. However, miR-10b is elevated in Huntington’s disease (HD) brains, and reduced levels of miR-10b associated with later age of motor onset in HD^67,68^. miR-24-1 was elevated 42% by Li treatment. This miRNA was downregulated in CSF samples of PD subjects^69^. It was also significantly reduced in CSF of AD vs. Control subjects^70^. miR-24 serves to reduce levels of Aβ through regulating expression of nicastrin^71^. Li regulation of glutamate metabotropic receptor 7 (GRM7) – a gene significantly associated in a GWAS study on bipolar patients – has been shown to act via miR-134^72,73^.

When we looked at potential networked effects for Li treatment vs. AD, we first note that none of the “core” AD genes we examined were altered by Li treatment in our experiment. However, when we examined both co-expression and gene product interaction, connections emerged that may be worthy of further investigation, particularly the interaction between SNORD14C and APP. Even though our Li treatment did not alter the levels of the core AD gene products, we did not test effects under more “pathogenic-like” conditions, such as oxidative stress, and it may be the differences between differences (non-stressed vs. stressed cells) that could further elucidate important pathways. Several of the connections we found in our network analysis, such as GSK3B, MAPT, ADAM17, and PSEN1 are also involved in other dementias than AD. However we hesitate to speculate on applicability of Li to multiple dementia disorders, since none of these were directly influenced by Li treatment in our study. Given that we restricted our work to a single differentiated cell line, we anticipate that much more can be learned by using other models, such as human primary brain cultures or induced pluripotent stem cell (iPSC) neuronal cultures.

Extending the role of Li treatment past bipolar disorder has revealed some challenges for its use. It is well known that, in higher doses, Li can be neurotoxic. It can suppress microtubule-associated protein tau and induce PD-like tremor and iron accumulation in brain^74^. Such iron accumulation is accompanied by reduction of levels of APP, which acts as an iron efflux protein^75^. Iron is only one of several metals that play a role in AD, in part due to the function of APP as an iron efflux protein^75^ that also responds to magnesium and lead^76,77^. Effective use of a metal as a drug may need to take into account interplay of multiple metals’ levels in the cells and environment.

Dosing “in the upper therapeutic range” (for bipolar disorder) can associate both with PD and AD-like symptoms and FDG-PET readouts, all of which were relieved by reduction of Li dose^78^. A larger-scale (>50,000 cases) retrospective study of Taiwanese patients administered Li reported mixed results^5^. On the other hand. low/micro-dose treatments improved agitation and stabilized cognitive impairment in AD^79,80^. When microdose Li was applied to animal AD models, it prevented memory loss and AD-like pathology^81^ as well as improved spatial memory and reduced Aβ42-induced neuroinflammation^82^.

Specific mechanisms of Li activity on AD may be explained by examining some of the Li-influenced coding gene products we mention herein. For example, BMP4 reduces hippocampal cell proliferation in animal AD models^83^, and BMP4 is elevated in AD^84^. Furthermore, APP regulates BMP/SMAD signaling in glial differentiation^85^. Another gene strongly implicated in AD is PPARγ, whose AD-related activities include possible inhibition of β-site amyloid cleaving enzyme 1 (BACE1)^86^ and SNP-based interaction with apolipoprotein E to alter AD risk^87^, among many other possible connections. Neurexin 3 (NRXN3) contains a polymorphism (rs17757879) that protects against AD in male subjects^88^. Furthermore, toxic Aβ oligomers interact with neurexins and reduce NRXN-mediated excitatory presynaptic organization^89^. Finally, NRXN3 is processed by the same α- and γ- secretases that produce the neurotrophic products of the “non-amyloidogenic” APP processing pathway^90^, including sAPPα. Other gene products we identified have their own potential associations with AD, as well, such as multiple snoRNAs that vary according to Braak stage^42^ and were also altered by Li-treatment in our study.

Our work herein highlights specific RNA species-specific responses to Li treatment, which would fuel mechanistic studies. While countless *in silico* pathways could have been found by examination of multiple databases, any claims connecting Li, snoRNAs, and disease states on that basis would be speculative. What we have experimentally discovered is that Li treatment has (at least) two distinct effects on RNA levels. The first is an RNA species-independent inverse relationship between RNA length and Li influence on RNA levels. The second is different effect levels by RNA type, irrespective of RNA length. The presence of miRNA as the least-influenced RNA type is particularly interesting, since it is in stark contrast against the majority of short RNA species. A different approach to investigating miRNA vs. Li treatment further underscored such “resistance” of miRNA to Li. When Li treatment response phenotypes in bipolar patients was screened by a large (1,693 sequence) genome-wide association study of pre-miRNA genes plus 20 kb flanking sequence, only one pre-miRNA gene had a nominally significant (p ≤ 0.05) sequence variation association, but when corrected for multiple comparisons, no polymorphisms in pre-miRNA plus flanking sequence showed any association with Li treatment phenotypes^91^. That study compared symptomatic Li response in patients to genetic sequences rather than response in cell culture transcriptome levels. This is not necessarily in conflict with known associations between miRNAs and bipolar disorder risk, since risk, progression, and treatment response may each be governed by distinct pathways.

 Such differences may suggest potentially useful avenues for investigating short RNA species in the multiple disorders currently and potentially treated by Li. What mechanisms could make miRNA less susceptible to Li treatment than RNA types of similar length? Are there genetic variations in miRNA response to Li, and do these correspond to Li treatment resistance or facilitation in patients? Analogous converse questions could be asked regarding snoRNAs and Li. We must point out that our study was on “steady state” RNA levels after one week of Li treatment.

While effects of Li on RNA levels were previously reported^32–35^, our study differs from these prior works in one critical and significant way. The preceding studies were limited by using microarray profiling. While the scope of microarrays has become quite impressive, they still each have a fixed number (albeit potentially thousands) of *pre-defined* targets. Our survey was high-throughput *sequencing* with no *a-priori* presumptions of the presence (or absence) of any specific RNA transcript.

We finally need to note that, while ATRA-induced SK-N-SH cultures are often accepted as “neuronal”, they are not adult human neurons. Further uses of non-presumptive whole-transcriptome sequencing follow-up to Li treatment should be done in other neuronal cell lines and in systems such as neuronal and mixed neuron/glial induced pluripotent stem cell culture, at the very least. Unfortunately, human neuronal cell models are always approximations of the actual human brain, as are any animal models. ATRA-induced SK-N-SH cultures have important elements of reproducibility and availability, which allows for seminal discoveries to be confirmed and later tested in the more costly and difficult models.

## Methods

### Oversight and approval

All procedures were approved and overseen by the Institutional Biosafety Committee, Office of Research Compliance, Indiana University, and Indianapolis, Indiana, USA.

### Cell culture and treatment

Human neuroblastoma (SK-N-SH) cells were obtained from ATCC and cultured as previously described^92^, then differentiated by ATRA^93^. Initial propagation was in Eagle’s modified minimum essential medium (MEM, ThermoFisher, Waltham MA) supplemented with 10% fetal bovine serum (FBS). For neuronal differentiation, a stock solution of 0.01 M ATRA (Sigma Prod. No. R2625; >98% HPLC purified), was prepared in absolute ethanol and stored in light protected vials at −20 °C and diluted with tissue culture medium right before use. Subsequent dilutions were made in growth medium with a final ethanol concentration of 0.1% (v/v) which did not affect the described system. During differentiation, cells were switched to MEM media with 1% FBS supplemented with 10 µM ATRA for two weeks. Cultures of treated SK-N-SH cells were subsequently treated with 1 mM Li chloride (LiCl) or vehicle (distilled water) for an additional week (n = 6 in each group). This dose is within a range of reported Li treatment of neuroblastoma cells characterized as “low” concentration^94,95^. Toxicity of Li treatment of neuroblastoma cells was reported to not occur until concentrations exceeded 10 mM^96^.

### RNA extraction and sequencing

Total RNA was extracted using the RNeasy mini kit (Qiagen Company, Germantown, MD) and ribosomal RNA depleted with Ribo-Zero (Illumina, San Diego CA). Whole transcriptome sequencing was done via SOLiD RNA-Seq kit (ThermoFisher). Briefly, total RNA was fragmented with RNase III, hybridized and ligated to SOLiD^TM^ adapters, then subject to reverse transcription with SOLiD^TM^ RT primer mix. cDNA was then amplified and sequenced on a 5500xl Genetic Analyzer (ThermoFisher). Identified transcripts were classified into RNA types using the typology and databases at, specifically pseudogene.org^97^, HGNC^98^, and GeneCards^99^.

### Statistical analysis

We analyzed quantitative transcriptome sequencing by edgeR^100^, which explicitly calculated fold differences in specific RNA levels between Li-treated and untreated cells and determined significance (p values and FDR) for each difference. We considered those RNAs that had level differences associated with FDR ≤ 0.2 to be “Li-influenced”, regardless of level of influence. We used the mclust^43^ package to explore possible clustered distribution of sequence lengths. Clustering with mclust used a univariate, unequal variances model to determine cluster borders and cluster centers were not predetermined.

RNA sequences were typologically classified by referring to three databases, specifically pseudogene.org^97^, HGNC^98^, and GeneCards^99^. The presence/absence of Li influence (significant change in RNA level in Li-treated vs. control cells) was determined by edgeR and coded as 1 or 0, 1 corresponding to Li-induced net RNA level change (either increase or decrease), by individual sequence. Magnitude of change was not considered in this study. Examination of the data showed that only 1.3% of sequences’ expression were influenced by Li treatment. Such a low frequency can introduce unacceptable bias into conventional logistic regression, which can be corrected by penalized log-likelihood estimation, such as via the logistf R package^101^. However, when we compared conventional and penalized logistic models for bias, we found that conventional methods produced no greater bias than did conventional regression for our data. Final analysis was, therefore, done with conventional logistic regression. A full model of “Li influenced ~ log(Length in bp) + RNA type + log(Length) × RNA type was constructed and assessed by second-order Akaike information criterion (AICc) using exhaustive combinations of predictors^102^ via the MuMIn R package^102^. Models were run with Effect contrast coding to explicitly compare Li influence of RNA species vs. the overall mean of Li influence frequency^103^. The effect coded model was further used for pairwise comparisons of RNA species by estimated marginal means^104^. Finally, the direction of Li-induced alteration (elevation, no change, or reduction of level) was modeled by multinomial logistic regression. Network analysis on selected coding gene products was performed vs. human hippocampus co-expression^105^ or protein-protein interaction^106^ dataset by NetworkAnalyst^107^, using the “minimum network” algorithm. Gene ontologies were analyzed by the “Gene Ontology System”^108^. For these utilities, default parameters were otherwise used.

### Ethical approval and informed consent

All procedures were approved and overseen by the Institutional Biosafety Committee (IBC), Office of Research Compliance, Indiana University, and Indianapolis, Indiana, USA. No animals or human subjects or samples were used in this work.

## Supplementary information

Supplementary Info

## Data Availability

Authors agree to make materials, data and associated protocols promptly available to readers without undue qualifications in material transfer agreements. Raw RNA sequence data is in process of deposition in GEO, accession # pending.
