# Supplementary material for: Lithium alters expression of RNAs in a type-specific manner in differentiated human neuroblastoma neuronal cultures, including specific genes involved in Alzheimer’s disease
Source: Sci Rep. 2019 Dec 4;9:18261. doi: 10.1038/s41598-019-54076-3 (PMC6892907; doi:10.1038/s41598-019-54076-3)
Supplement: Supplementary file 1 — Supplementary Info [file 41598_2019_54076_MOESM1_ESM.pdf]

Lithium alters expression of RNAs in a type-specific manner in differentiated human neuroblastoma neuronal cultures, including specific genes involved in Alzheimer's disease

Bryan Maloney, Yokesh Balaraman, Yunlong Liu, Nipun Chopra, Howard J Edenberg, John Kelsoe, John I Nurnberger and Debomoy K Lahiri

Supplementary tables 1-4

Supplemental table 1: RNA transcripts with expression altered by lithium treatment (FDR ≤ 0.2)

Gene	Name	Change	p	FDR
AGAP9_dup1	ArfGAP with GTPase domain, ankyrin repeat and PH domain 9	−84%	< 0.0001	< 0.0001
MIR6516	miR-6516	+114%	< 0.0001	< 0.0001
SNORA23	Small Nucleolar RNA, H/ACA box 23	+144%	< 0.0001	< 0.0001
SNORA44	Small Nucleolar RNA, H/ACA box 44	+125%	< 0.0001	< 0.0001
SNORA45B	Small Nucleolar RNA, H/ACA box 45B	+107%	< 0.0001	< 0.0001
SNORA80E	Small Nucleolar RNA, H/ACA box 80E	+234%	< 0.0001	< 0.0001
SNORD100	Small Nucleolar RNA, C/D box 100	+98%	< 0.0001	< 0.0001
SNORD104	Small Nucleolar RNA, C/D box 104	+84%	< 0.0001	< 0.0001
SNORD31	Small Nucleolar RNA, C/D box 31	+100%	< 0.0001	< 0.0001
SNORD44	Small Nucleolar RNA, C/D box 44	+90%	< 0.0001	< 0.0001
SNORD69	Small Nucleolar RNA, C/D box 69	+122%	< 0.0001	< 0.0001
SNORD9	Small Nucleolar RNA, C/D box 9	+132%	< 0.0001	< 0.0001
SNORD92	Small Nucleolar RNA, C/D box 92	+107%	< 0.0001	< 0.0001
SNORD95	Small Nucleolar RNA, C/D box 95	+76%	< 0.0001	< 0.0001
SCARNA1	Small Cajal body-specific RNA 1	+118%	< 0.0001	0.0002
SNORD27	Small Nucleolar RNA, C/D box 27	+79%	< 0.0001	0.0002
SNORD42A	Small Nucleolar RNA, C/D box 42A	+61%	< 0.0001	0.0002
SNORD83B	Small Nucleolar RNA, C/D box 83B	+130%	< 0.0001	0.0004
RNU4ATAC	RNA, U4atac Small Nuclear (U12-Dependent Splicing)	+89%	< 0.0001	0.0009
SNORA67	Small Nucleolar RNA, H/ACA box 67	+76%	< 0.0001	0.0009
SNORD99	Small Nucleolar RNA, C/D box 99	+82%	< 0.0001	0.0009
SNORD71	Small Nucleolar RNA, C/D box 71	+82%	< 0.0001	0.0010
SNORD83A	Small Nucleolar RNA, C/D box 83A	+66%	< 0.0001	0.0014
SNORA21	Small Nucleolar RNA, H/ACA box 21	+76%	< 0.0001	0.0019
SNORD96A	Small Nucleolar RNA, C/D box 96A	+66%	< 0.0001	0.0019
SNORD41	Small Nucleolar RNA, C/D box 41	+45%	< 0.0001	0.0024
SNORD12B	Small Nucleolar RNA, C/D box 12B	+73%	< 0.0001	0.0026
SCARNA3	Small Cajal body-specific RNA 3	+93%	< 0.0001	0.0039
SNORD87	Small Nucleolar RNA, C/D box 87	+66%	< 0.0001	0.0040
SNORD5	Small Nucleolar RNA, C/D box 5	+55%	< 0.0001	0.0054
SNORA4	Small Nucleolar RNA, H/ACA box 4	+68%	< 0.0001	0.0058
SNORD55	Small Nucleolar RNA, C/D box 55	+43%	< 0.0001	0.0065
SNORD42B	Small Nucleolar RNA, C/D box 42B	+52%	< 0.0001	0.0089
SNORD116-29	Small Nucleolar RNA, C/D box 116-29	+74%	< 0.0001	0.0093
SNORA80A	Small Nucleolar RNA, H/ACA box 80A	+62%	< 0.0001	0.0107
KCNH4	Potassium Voltage-Gated Channel Subfamily H Member 4	−37%	< 0.0001	0.0109
SNORD114-26	Small Nucleolar RNA, C/D box 114-26	+125%	< 0.0001	0.0115
SNORD18A	Small Nucleolar RNA, C/D box 18A	+51%	< 0.0001	0.0123
SNORD53	Small Nucleolar RNA, C/D box 53	+50%	< 0.0001	0.0135
SNORD78	Small Nucleolar RNA, C/D box 78	+62%	< 0.0001	0.0135
PET100	PET100 Homolog	+45%	< 0.0001	0.0155
RPL23A	Ribosomal Protein L23	+45%	< 0.0001	0.0177

Gene	Name	Change	p	FDR
SNORA76A	Small Nucleolar RNA, H/ACA box 76A	+66%	< 0.0001	0.0177
RNU12	RNA, U12 Small Nuclear	+118%	< 0.0001	0.0178
SNORD57	Small Nucleolar RNA, C/D box 57	+59%	< 0.0001	0.0178
SNORD8	Small Nucleolar RNA, C/D box 8	+66%	< 0.0001	0.0187
GSTZ1	Glutathione S-Transferase Zeta 1	+36%	< 0.0001	0.0189
BMS1P6_dup1	BMS1, ribosome biogenesis factor pseudogene 6	−64%	< 0.0001	0.0206
SNORD73A	Small Nucleolar RNA, C/D box 73A	+90%	< 0.0001	0.0206
SNORA14A	Small Nucleolar RNA, H/ACA box 14A	+78%	< 0.0001	0.0227
HIST3H2BB	Histone Cluster 3 H2B Family Member B	+61%	< 0.0001	0.0239
SNORD34	Small Nucleolar RNA, C/D box 34	+54%	< 0.0001	0.0252
SNORA14B	Small Nucleolar RNA, H/ACA box 14B	+40%	< 0.0001	0.0287
SNORA52	Small Nucleolar RNA, H/ACA box 52	+53%	0.0001	0.0293
SNORA62	Small Nucleolar RNA, H/ACA box 62	+64%	0.0001	0.0293
POM121	Nuclear envelope pore membrane protein POM 121	−39%	0.0001	0.0327
SNORD22	Small Nucleolar RNA, C/D box 22	+73%	0.0001	0.0347
SNORA2A	Small Nucleolar RNA, H/ACA box 2A	+71%	0.0001	0.0358
SNORD35A	Small Nucleolar RNA, C/D box 35A	+52%	0.0001	0.0358
CBLN2	Cerebellin 2 Precursor	+47%	0.0001	0.0368
SNORA26	Small Nucleolar RNA, H/ACA box 26	+46%	0.0002	0.0389
SNORA6	Small Nucleolar RNA, H/ACA box 6	+62%	0.0002	0.0394
CUX2	Cut Like Homeobox 2	−27%	0.0002	0.0397
PWAR4	Prader Willi/Angelman Region RNA 4	+86%	0.0002	0.0397
SNORA53	Small Nucleolar RNA, H/ACA box 53	+61%	0.0002	0.0397
MIR4644	miR-4644	−50%	0.0002	0.0407
SNHG7	Small Nucleolar RNA Host Gene 7	+49%	0.0002	0.0416
SNORA17	Small Nucleolar RNA, H/ACA box 17	+57%	0.0002	0.0416
SCARNA22	Small Cajal body-specific RNA 22	+42%	0.0002	0.0417
DNAJC9	DnaJ Heat Shock Protein Family (Hsp40) Member C9	+37%	0.0002	0.0425
SCARNA6	Small Cajal body-specific RNA 6	+53%	0.0002	0.0425
SNORD67	Small Nucleolar RNA, C/D box 67	+75%	0.0002	0.0425
RPS10P7	Ribosomal Protein S10 Pseudogene 7	−56%	0.0002	0.0434
GPR112	G protein-coupled receptor 112	+215%	0.0002	0.0440
SNORD46	Small Nucleolar RNA, C/D box 46	+48%	0.0002	0.0464
HIST1H2BE	Histone Cluster 1 H2B Family Member E	+41%	0.0002	0.0469
SNORD79	Small Nucleolar RNA, C/D box 79	+73%	0.0003	0.0501
SNORD115-23	Small Nucleolar RNA, C/D box 115-23	+84%	0.0003	0.0502
CRIPAK	cysteine-rich PAK1 inhibitor	−58%	0.0003	0.0512
SNORD88C	Small Nucleolar RNA, C/D box 88C	+62%	0.0003	0.0590
ANO3	Anoctamin-3	+41%	0.0003	0.0593
SNORA37	Small Nucleolar RNA, H/ACA box 37	+70%	0.0003	0.0593
DCLK3	Serine/threonine-protein kinase	+48%	0.0003	0.0629
HGF	Hepatocyte Growth Factor	+38%	0.0004	0.0649
SNORD26	Small Nucleolar RNA, C/D box 26	+56%	0.0004	0.0662
ARHGAP36	Rho GTPase activating protein 36	+29%	0.0004	0.0667
TIMM8A	Mitochondrial import inner membrane translocase subunit Tim8 A	−36%	0.0004	0.0667
SCARNA11	Small Cajal body-specific RNA 11	+44%	0.0004	0.0688

Gene	Name	Change	p	FDR
SNORA11	Small Nucleolar RNA, H/ACA box 11	+52%	0.0004	0.0688
SNORA43	Small Nucleolar RNA, H/ACA box 43	+55%	0.0004	0.0751
GPRC5B	G Protein-Coupled Receptor Class C Group 5 Member B	−34%	0.0005	0.0768
NMB	Neuromedin B	+41%	0.0005	0.0768
PIANP	PILR Alpha Associated Neural Protein	−65%	0.0005	0.0768
SYTL4	Synaptotagmin Like 4	+33%	0.0005	0.0768
ZIK1	Zinc Finger Protein Interacting With K Protein 1	+27%	0.0005	0.0768
SNORD33	Small Nucleolar RNA, C/D box 33	+31%	0.0005	0.0781
HIST2H2BE	histone H2B type 2-E	+37%	0.0005	0.0814
SNORA45A	Small Nucleolar RNA, H/ACA box 45A	+61%	0.0005	0.0836
HS6ST2	Heparan Sulfate 6-O-Sulfotransferase 2	+41%	0.0006	0.0858
SNORA55	Small Nucleolar RNA, H/ACA box 55	+46%	0.0006	0.0872
SNORD111B	Small Nucleolar RNA, C/D box 111B	+48%	0.0006	0.0891
ARPC5L	actin related protein 2/3 complex subunit 5 like	+34%	0.0006	0.0899
DPT	Dermatopontin	+46%	0.0006	0.0902
LOC157273	Uncharacterized LOC157273	−27%	0.0006	0.0909
NBPF25P_dup3	Neuroblastoma Breakpoint Family Member 25, Pseudogene	−57%	0.0006	0.0909
SNORD74	Small Nucleolar RNA, C/D box 74	+90%	0.0006	0.0909
ZNF433	Zinc Finger Protein 433	+33%	0.0006	0.0909
SNORA25	Small Nucleolar RNA, H/ACA box 25	+59%	0.0006	0.0920
SLC25A43	Solute carrier family 25 member 43	+72%	0.0007	0.0920
BMP4	Bone Morphogenetic Protein 4	+51%	0.0007	0.0932
SCARNA18	Small Cajal body-specific RNA 18	+93%	0.0007	0.0932
NBPF9_dup2	Neuroblastoma Breakpoint Family Member 9	−55%	0.0007	0.0941
UTRN	Utrophin	+36%	0.0007	0.0941
NRXN3	Neurexin 3	−43%	0.0007	0.0957
ABHD4	Abhydrolase domain containing 4	−29%	0.0007	0.0960
NBPF14	Neuroblastoma Breakpoint Family Member 14	−64%	0.0007	0.0960
USH2A	Usherin	−36%	0.0007	0.0960
SCARNA9	Small Cajal body-specific RNA 9	+57%	0.0007	0.0976
GRIA3	Glutamate receptor 3	−27%	0.0008	0.0976
SCARNA16	Small Cajal body-specific RNA 16	+63%	0.0008	0.0991
SNORD94	Small Nucleolar RNA, C/D box 94	+63%	0.0008	0.0991
NPIPB3	Nuclear Pore Complex Interacting Protein Family Member B3	−52%	0.0008	0.1011
SNORA71D	Small Nucleolar RNA, H/ACA box 71D	+65%	0.0008	0.1011
YAP1	Yes Associated Protein 1	−32%	0.0008	0.1017
SNORD89	Small Nucleolar RNA, C/D box 89	+44%	0.0009	0.1070
RPS28	Ribosomal Protein S28	−40%	0.0009	0.1085
IGFBP2	Insulin Like Growth Factor Binding Protein 2	+28%	0.0010	0.1163
DDX39A	DEAD (Asp-Glu-Ala-Asp) box polypeptide 39A	+23%	0.0010	0.1166
PRC1	Protein Regulator Of Cytokinesis 1	+26%	0.0010	0.1166
RAB27B	Ras-related protein Rab-27B	+28%	0.0010	0.1166
TFAMP1	Transcription Factor A, Mitochondrial Pseudogene 1	−23%	0.0010	0.1166
WIBG	PYM homolog 1, exon junction complex associated factor	+28%	0.0010	0.1168

Gene	Name	Change	p	FDR
IRS1	Insulin Receptor Substrate 1	−28%	0.0010	0.1180
MIR3651	miR-3651	+55%	0.0010	0.1180
SNORA84	Small Nucleolar RNA, H/ACA box 84	+55%	0.0010	0.1180
GLIS3	GLI-Similar Family Zinc Finger 3	−42%	0.0011	0.1190
SNORD24	Small Nucleolar RNA, C/D box 24	+56%	0.0011	0.1190
GGN	Gametogenetin	−42%	0.0011	0.1195
SNORA11B	Small Nucleolar RNA, H/ACA box 11B	+90%	0.0011	0.1195
ACAA1	3-Ketoacyl-CoA thiolase, peroxisomal precursor	−32%	0.0011	0.1204
SNORD35B	Small Nucleolar RNA, C/D box 35B	+33%	0.0011	0.1204
C9orf24	Chromosome 9 Open Reading Frame 24	−34%	0.0011	0.1219
GPSM2	G-Protein Signaling Modulator 2	+25%	0.0011	0.1219
ZWINT	ZW10 Interacting Kinetochore Protein	+27%	0.0011	0.1219
RASEF	Ras-related protein Rab-45	+35%	0.0012	0.1261
CCNF	G2/mitotic-specific cyclin-F	+25%	0.0013	0.1324
SCUBE1	Signal Peptide, CUB Domain And EGF Like Domain Containing 1	−29%	0.0013	0.1324
SNORA80B	Small Nucleolar RNA, H/ACA box 80B	+61%	0.0013	0.1324
SNORD45A	Small Nucleolar RNA, C/D box 45A	+44%	0.0014	0.1400
PAPPA2	Pappalysin 2	+37%	0.0014	0.1467
HMGB2	High Mobility Group Box 2	+25%	0.0015	0.1496
PTMA	Prothymosin, Alpha	+48%	0.0015	0.1496
FAM222B	family with sequence similarity 222 member B	−42%	0.0015	0.1542
SNORD18B	Small Nucleolar RNA, C/D box 18B	+48%	0.0016	0.1557
VTRNA1-3	Vault RNA 1-3	+53%	0.0016	0.1557
SNORD60	Small Nucleolar RNA, C/D box 60	+42%	0.0016	0.1570
INPP5J	Inositol Polyphosphate-5-Phosphatase J	−30%	0.0016	0.1577
MIR1291	miR-1291	+48%	0.0016	0.1577
SCARNA13	Small Cajal body-specific RNA 13	+36%	0.0016	0.1577
GRIN2D	Glutamate Ionotropic Receptor NMDA Type Subunit 2D	−57%	0.0017	0.1586
NATD1	N-Acetyltransferase Domain Containing 1	−46%	0.0017	0.1586
BCYRN1	Brain Cytoplasmic RNA 1	−37%	0.0017	0.1602
MIR24-1	miR-24-1	+42%	0.0017	0.1602
SNORD59B	Small Nucleolar RNA, C/D box 59B	+42%	0.0017	0.1602
PAPPA-AS1	PAPPA Antisense RNA 1	−48%	0.0018	0.1642
SNORD47	Small Nucleolar RNA, C/D box 47	+37%	0.0018	0.1647
SNRNP25	U11/U12 small nuclear ribonucleoprotein 25 kDa protein	+28%	0.0018	0.1647
SDHC	Succinate Dehydrogenase Complex Subunit C	−35%	0.0018	0.1656
SNORA34	Small Nucleolar RNA, H/ACA box 34	+48%	0.0018	0.1667
GRINA	Glutamate Ionotropic Receptor NMDA Type Subunit Associated Protein 1	−27%	0.0019	0.1709
RAP2C-AS1	RAP2C Antisense RNA 1	−36%	0.0019	0.1709
RTBDN	Retbindin	−47%	0.0019	0.1709
SNORD29	Small Nucleolar RNA, C/D box 29	+50%	0.0019	0.1709
SNHG10	Small Nucleolar RNA Host Gene 10	+35%	0.0019	0.1722
IGLL5	Immunoglobulin Lambda Like Polypeptide 5	−42%	0.0020	0.1755
LINC01359	Long Intergenic Non-Protein Coding RNA 1359	−36%	0.0021	0.1801
CTC-338M12.4	Uncharacterized LOC101928649	−33%	0.0021	0.1807

Gene	Name	Change	p	FDR
MIDN	Midnolin	−47%	0.0021	0.1807
MIR641	miR-641	−32%	0.0021	0.1807
NLGN3	Neuroligin 3	−37%	0.0021	0.1807
SMAD6	SMAD Family Member 6	−29%	0.0021	0.1807
SNORA56	Small Nucleolar RNA, H/ACA box 56	+37%	0.0021	0.1807
PPARG	Peroxisome proliferator-activated receptor gamma	−26%	0.0022	0.1807
SOX5	SRY-Box 5	−45%	0.0022	0.1807
BREA2	Breast Cancer Estrogen-Induced Apoptosis 2	−57%	0.0022	0.1824
CXorf49_dup1	Chromosome X Open Reading Frame 49	−37%	0.0022	0.1824
FBXL16	F-box and leucine-rich repeat protein 16	−50%	0.0022	0.1824
RAB3A	Ras-related protein Rab-3A	−44%	0.0022	0.1824
ZMYM3	Zinc Finger MYM-Type Containing 3	−25%	0.0022	0.1824
CXorf49B_dup1	Chromosome X Open Reading Frame 49B	−37%	0.0023	0.1824
GREM2	Gremlin-2 Precursor	−28%	0.0023	0.1846
MAFB	MAF BZIP Transcription Factor B	−36%	0.0023	0.1846
MIR4803	miR-4803	−32%	0.0023	0.1846
OCLM	Oculomedin	−40%	0.0023	0.1846
TICAM1	Toll Like Receptor Adaptor Molecule 1	−38%	0.0023	0.1846
PHYHIPL	Phytanoyl-CoA 2-Hydroxylase Interacting Protein Like	−21%	0.0024	0.1852
ARGLU1	Arginine and glutamate-rich protein 1	+25%	0.0024	0.1904
LINC01252	Long Intergenic Non-Protein Coding RNA 1252	−44%	0.0024	0.1904
HIST2H3D	Histone Cluster 2 H3D	+45%	0.0025	0.1921
MIR10B	miR-10b	+44%	0.0025	0.1921
SEMA3D	Semaphorin 3D	−27%	0.0025	0.1921
SNORD91B	Small Nucleolar RNA, C/D box 91B	+49%	0.0025	0.1929
MANBAL	Mannosidase B Like	−27%	0.0025	0.1931
PRR12	Proline Rich 12	−53%	0.0026	0.1935
FMNL1	Formin-like protein 1	−37%	0.0026	0.1947
TGFB1I1	Transforming Growth Factor B 1 Induced Transcript 1	−24%	0.0026	0.1947
SMS	Spermine Synthase	+31%	0.0026	0.1950

Supplemental table 2

Distribution of magnitudes of Li-induced alterations to RNA levels (FDR ≤ 0.2)

RNA Level Change		Total RNAs
log2	Percent	
−2.5 to −2.3	−82% to −80%	1
−2.3 to −2.1	−80% to −77%	0
−2.1 to −1.9	−77% to −73%	0
−1.9 to −1.7	−73% to −69%	0
−1.7 to −1.5	−69% to −65%	0
−1.5 to −1.3	−65% to −59%	1
−1.3 to −1.1	−59% to −53%	2
−1.1 to −0.9	−53% to −46%	6
−0.9 to −0.7	−46% to −38%	7
−0.7 to −0.5	−38% to −29%	12
−0.5 to −0.3	−29% to −19%	21
−0.3 to −0.1	−19% to −7%	16
−0.1 to +0.1	−7% to +7%	0
+0.1 to +0.3	+7% to +23%	0
+0.3 to +0.5	+23% to +41%	0
+0.5 to +0.7	+41% to +62%	35
+0.7 to +0.9	+62% to +87%	56
+0.9 to +1.1	+87% to +114%	28
+1.1 to +1.3	+114% to +146%	12
+1.3 to +1.5	+146% to +183%	8
+1.5 to +1.7	+183% to +225%	0
+1.7 to +1.9	+225% to +273%	1
+1.9 to +2.1	+273% to +329%	1
+2.1 to +2.3	+329% to +392%	0
+2.3 to +2.5	+392% to +466%	0

Supplemental table 3

Figure Symbol	Gene Symbol
co1	AGTRAP
co2	ATF6
co3	ATP2A2
co4	BACH1
co5	BHLHE22
co6	CD63
co7	CLCN4
co8	CLVS1
co9	CMTM6
co10	COPG1
co11	CWF19L2
co12	DCTN2
co13	DLGAP1
co14	DYNC1LI1
co15	DYNLT1
co16	EFEMP1
co17	EIF3G
co18	FAM65B
co19	FRMPD4
co20	GOT1
co21	GRHPR
co22	HIVEP2
co23	ILF2
co24	LAMP2
co25	LMAN1
co26	LMTK2
co27	MPP5
co28	NMNAT2
co29	NUP160
co30	PAK2
co31	PDPN
co32	PI4KA
co33	PLEKHB2
co34	PPM1H
co35	PTPN21
co36	RALYL
co37	REEP1
co38	RNF19A
co39	SERPINB6
co40	SF3B1
co41	SIK2
co42	SIRPA
co43	SLC41A1
co44	SPRYD7
co45	SPTLC1

co46	SSR1
co47	STXBP3
co48	TRAFD1
co49	TRAM1
co50	TTYH2
co51	UBE2A
co52	UBE4A
co53	USO1
co54	VPS18
co55	YIPF2
co56	YWHAG
co57	ZNF217

Supplemental table 4

Figure Symbol	Gene Symbol
in1	ACTB
in2	ACTB
in3	ADRB2
in4	AKT1
in5	APOE
in6	AR
in7	AURKA
in8	BMPR1B
in9	BRCA1
in10	CALM1
in11	CBX8
in12	CD81
in13	CDK17
in14	CENPB
in15	CLEC3B
in16	CNBP
in17	COPS5
in18	CTNNB1
in19	CUL3
in20	CUL7
in21	EEF1A1
in22	EGFR
in23	EIF3F
in24	EIF4A3
in25	EIF4E
in26	EPB41L3
in27	ERG
in28	ESR1
in29	FBXW7
in30	FUS
in31	GSDMB
in32	HDAC5
in33	HSPA4
in34	HSPB1
in35	IFIH1
in36	IGF1R
in37	ITGB1
in38	LOC100129518
in39	MAPK1
in40	MTOR
in41	MYC
in42	NCOR1
in43	NEDD4
in44	NUMB
in45	NXF1

in46	P4HB
in47	PAN2
in48	PARK2
in49	PAXIP1
in50	PDIA3
in51	PML
in52	PRKCD
in53	PTN
in54	RNF2
in55	RPL23
in56	RPL30
in57	SH3GL2
in58	SIN3A
in59	SIRT7
in60	SMAD3
in61	SMAD7
in62	SOCS3
in63	SOX2
in64	STAU1
in65	TGFBR1
in66	TP63
in67	TRAF6
in68	TSPAN5
in69	UBC
in70	YWHAZ
